# Supplementary material for: Effect of Sirolimus vs. Everolimus on CMV-Infections after Kidney Transplantation—A Network Meta-Analysis
Source: J Clin Med. 2022 Jul 20;11(14):4216. doi: 10.3390/jcm11144216 (PMC9323040; doi:10.3390/jcm11144216)
Supplement: Supplementary file 1 [file jcm-11-04216-s001.zip › Table S1.pdf]

Table S1. SIR vs. CNI<br><br>Trial	Therapy	Induction	mTOR-I (pts.)	all CNI (pts.)	mTOR-I initiation	Duration of study (month)	Follow-Up longterm (month)	CMV 12 months post Tx(%)	ITT
Buchler 2007, Lebranchu 2012, Gatault 2016 (1-3) (RTx)	Sir<br>CsA	ATG<br>ATG	71	 74	de novo	12	60 (96)	6<br>23	YES
Cianco 2004, Guerra 2011 (4-6) (RTx)	Sir+CsA<br>Sir+Tac<br>Tac+MPA	Daclizumab<br>Daclizumab<br>Daclizumab	50<br>50	 <br>50	de novo	12	96	0<br>0<br>4	YES
Durrbach 2008 (7) RTx	Sir+MPA<br>CsA+MPA	ATG<br>ATG	33	 36	de novo	6	-	0<br>11	YES
Ekberg 2007, Ekberg 2009 (8, 9) (RTx)	Standard CsA<br>Low CsA<br>Low Tac<br>Low Sir	None<br>Daclizumab<br>Daclizumab<br>Daclizumab	 <br><br>399	1190	de novo	12	36	14,3<br>11,0<br>9,7<br>6,1	YES
Glotz 2010 (10) (RTx)	Sir+MPA<br>Tac+MPA	ATG<br>ATG	71	 70	de novo	12	-	1,4<br>20	YES
Guba 2010/2012 (11, 12) (RTx)	Sir<br>CsA	ATG-F<br>ATG-F	69	 71	14-21 d post Tx	12	36	7,3<br>28,2	YES
Huh 2017(13) (RTx)	Sir+Tac<br>Tac+MPA	Basiliximab<br>Basiliximab	76	 75	de novo	12	-	1,3<br>9,3	YES
Kandaswamy 2005, Suszynski 2013 (14, 15) (RTx)	CsA + MMF<br>hi TAC + SIR<br>lo TAC + SIR	ATG<br>ATG<br>ATG	 72 (145)<br>82 (134)	85 (146)	de novo	12	24	5 (8,4)<br>5 (6,9)<br>4 (4,5)	YES
Lebranchu 2009, Servais 2009, Lebranchu 2011 (16-18) (RTx)	Sir<br>CsA	Daclizumab<br>Daclizumab	95	 97	3 mo post Tx	12	48	4<br>6	YES
Rummo 2017, Rummo 2020 (19, 20) (RTx)	Sir+Tac<br>Tac+MPA	n.r.	282	 287	28 d post Tx	12	60	3,8<br>11,9	YES
Sampaio 2008 (21) (RTx)	Sir+Tac<br>Tac+MPA	None	50	 50	de novo	12	-	12<br>12	YES
Van Gurp 2010 (22) (RTx)	Sir+Tac<br>Tac+MPA	Daclizumab<br>Daclizumab	318	 316	de novo	6	-	2,8<br>12,0	NO
Vitko 2006 (23) (RTx)	Tac+MPA<br>Tac+Sir 0,5<br>Tac+Sir 2,0	None	 325<br>325	327	de novo	6	-	8,0<br>4,9<br>4,0	YES

1. Buchler M, Caillard S, Barbier S*, et al*: Sirolimus versus cyclosporine in kidney recipients receiving thymoglobulin, mycophenolate mofetil and a 6-month course of steroids. Am J Transplant 7: 2522-2531, 2007.
2. Lebranchu Y, Snanoudj R, Toupance O*, et al*: Five-year results of a randomized trial comparing de novo sirolimus and cyclosporine in renal transplantation: the SPIESSER study. Am J Transplant 12: 1801-1810, 2012.
3. Gatault P, Bertrand D, Buchler M*, et al*: Eight-year results of the Spiesser study, a randomized trial comparing de novo sirolimus and cyclosporine in renal transplantation. Transpl Int 29: 41-50, 2016.
4. Ciancio G, Burke GW, Gaynor JJ*, et al*: A randomized long-term trial of tacrolimus/sirolimus versus tacrolimus/mycophenolate mofetil versus cyclosporine (NEORAL)/sirolimus in renal transplantation. II. Survival, function, and protocol compliance at 1 year. Transplantation 77: 252-258, 2004.

5. Ciancio G, Burke GW, Gaynor JJ*, et al*: A randomized long-term trial of tacrolimus and sirolimus versus tacrolimus and mycophenolate mofetil versus cyclosporine (NEORAL) and sirolimus in renal transplantation. I. Drug interactions and rejection at one year. Transplantation 77: 244-251, 2004.
6. Guerra G, Ciancio G, Gaynor JJ*, et al*: Randomized trial of immunosuppressive regimens in renal transplantation. J Am Soc Nephrol 22: 1758-1768, 2011.
7. Durrbach A, Rostaing L, Tricot L*, et al*: Prospective comparison of the use of sirolimus and cyclosporine in recipients of a kidney from an expanded criteria donor. Transplantation 85: 486-490, 2008.
8. Ekberg H, Tedesco-Silva H, Demirbas A*, et al*: Reduced exposure to calcineurin inhibitors in renal transplantation. N Engl J Med 357: 2562-2575, 2007.
9. Ekberg H, Bernasconi C, Tedesco-Silva H*, et al*: Calcineurin inhibitor minimization in the Symphony study: observational results 3 years after transplantation. Am J Transplant 9: 1876-1885, 2009.
10. Glotz D, Charpentier B, Abramovicz D*, et al*: Thymoglobulin induction and sirolimus versus tacrolimus in kidney transplant recipients receiving mycophenolate mofetil and steroids. Transplantation 89: 1511-1517, 2010.
11. Guba M, Pratschke J, Hugo C*, et al*: Renal function, efficacy, and safety of sirolimus and mycophenolate mofetil after short-term calcineurin inhibitor-based quadruple therapy in de novo renal transplant patients: one-year analysis of a randomized multicenter trial. Transplantation 90: 175-183, 2010.
12. Guba M, Pratschke J, Hugo C*, et al*: Early conversion to a sirolimus-based, calcineurin-inhibitor-free immunosuppression in the SMART trial: observational results at 24 and 36months after transplantation. Transpl Int 25: 416-423, 2012.
13. Huh KH, Lee JG, Ha J*, et al*: De novo low-dose sirolimus versus mycophenolate mofetil in combination with extended-release tacrolimus in kidney transplant recipients: a multicentre, open-label, randomized, controlled, non-inferiority trial. Nephrol Dial Transplant 32: 1415-1424, 2017.
14. Kandaswamy R, Melancon JK, Dunn T*, et al*: A prospective randomized trial of steroid-free maintenance regimens in kidney transplant recipients--an interim analysis. Am J Transplant 5: 1529-1536, 2005.
15. Suszynski TM, Gillingham KJ, Rizzari MD*, et al*: Prospective randomized trial of maintenance immunosuppression with rapid discontinuation of prednisone in adult kidney transplantation. Am J Transplant 13: 961-970, 2013.
16. Lebranchu Y, Thierry A, Toupance O*, et al*: Efficacy on renal function of early conversion from cyclosporine to sirolimus 3 months after renal transplantation: concept study. Am J Transplant 9: 1115-1123, 2009.
17. Servais A, Meas-Yedid V, Toupance O*, et al*: Interstitial fibrosis quantification in renal transplant recipients randomized to continue cyclosporine or convert to sirolimus. Am J Transplant 9: 2552-2560, 2009.
18. Lebranchu Y, Thierry A, Thervet E*, et al*: Efficacy and safety of early cyclosporine conversion to sirolimus with continued MMF-four-year results of the Postconcept study. Am J Transplant 11: 1665-1675, 2011.
19. Rummo OO, Carmellini M, Rostaing L*, et al*: ADHERE: randomized controlled trial comparing renal function in de novo kidney transplant recipients receiving prolonged-release tacrolimus plus mycophenolate mofetil or sirolimus. Transpl Int 30: 83-95, 2017.

20. Rummo O, Carmellini M, Kamar N*, et al*: Long-term, prolonged-release tacrolimus-based immunosuppression in de novo kidney transplant recipients: 5-year prospective follow-up of the ADHERE study patients. Transpl Int 33: 161-173, 2020.
21. Sampaio EL, Pinheiro-Machado PG, Garcia R*, et al*: Mycophenolate mofetil vs. sirolimus in kidney transplant recipients receiving tacrolimus-based immunosuppressive regimen. Clin Transplant 22: 141-149, 2008.
22. Van Gurp E, Bustamante J, Franco A*, et al*: Comparable Renal Function at 6 Months with Tacrolimus Combined with Fixed-Dose Sirolimus or MMF: Results of a Randomized Multicenter Trial in Renal Transplantation. J Transplant 2010: 2010.
23. Vitko S, Wlodarczyk Z, Kyllonen L*, et al*: Tacrolimus combined with two different dosages of sirolimus in kidney transplantation: results of a multicenter study. Am J Transplant 6: 531-538, 2006.
